# Distribution of Class 1 Integrons with IS*26*-Mediated Deletions in Their 3′-Conserved Segments in *Escherichia coli* of Human and Animal Origin

**DOI:** 10.1371/journal.pone.0012754

**Published:** 2010-09-15

**Authors:** Fay E. Dawes, Alexander Kuzevski, Karl A. Bettelheim, Michael A. Hornitzky, Steven P. Djordjevic, Mark J. Walker

**Affiliations:** 1 School of Biological Sciences, University of Wollongong, Wollongong, New South Wales, Australia; 2 Microbiological Diagnostic Unit, Department of Microbiology and Immunology, University of Melbourne, Parkville, Victoria, Australia; 3 Microbiology and Immunology Section, Industry and Investment NSW, Elizabeth Macarthur Agricultural Institute, Menangle, New South Wales, Australia; 4 The ithree Institute, University of Technology, Sydney, New South Wales, Australia; 5 School of Chemistry and Molecular Biosciences, University of Queensland, Brisbane, Queensland, Australia; Baylor College of Medicine, United States of America

## Abstract

Class 1 integrons play a role in the emergence of multi-resistant bacteria by facilitating the recruitment of gene cassettes encoding antibiotic resistance genes. 512 *E. coli* strains sourced from humans (n = 202), animals (n = 304) and the environment (n = 6) were screened for the presence of the *intI1* gene. In 31/79 integron positive *E. coli* strains, the gene cassette regions could not be PCR amplified using standard primers. DNA sequence analysis of 6 serologically diverse strains revealed atypical integrons harboured the *dfrA5* cassette gene and only 24 bp of the integron 3′-conserved segment (CS) remained, due to the insertion of IS*26*. PCR targeting *intI1* and IS*26* followed by restriction fragment length polymorphism (RFLP) analysis identified the integron-*dfrA5*-IS*26* element in 27 *E. coli* strains of bovine origin and 4 strains of human origin. Southern hybridization and transformation studies revealed the integron-*dfrA5*-IS*26* gene arrangement was either chromosomally located or plasmid borne. Plasmid location in 4/9 *E. coli* strains and PCR linkage of Tn*21* transposition genes with the *intI1* gene in 20/31 strains, suggests this element is readily disseminated by horizontal transfer.

## Introduction

The emergence and spread of antibiotic resistance is an escalating global health concern. Multi-drug resistant bacteria are the principal cause of failure in the treatment of infectious diseases, resulting in increases in the term and magnitude of morbidity, higher rates of mortality, and a greater health cost burden [1]. Monitoring and surveillance of antibiotic resistance genes in the community is essential for developing strategies to minimize the spread of antimicrobial resistance and evaluating their impact. Class 1 integrons play an important role in the emergence of multi-resistant bacteria via the stockpiling of resistance determinants [2], [3], [4]. Integrons, while not mobile themselves are often carried by mobilizing elements such as conjugative plasmids, transposons or phages, which ensure their horizontal transfer [5].

Class 1 integrons are usually comprised of two conserved segments, which flank the variable gene cassette region. Essential features of the 5′-CS are the integrase gene (*intI1*), the product of which mediates orientation specific integration and excision of gene cassettes [6], a promoter region, containing the potential promoter, Pc to allow expression of the inserted cassette genes [7], and an integration site (*attI1*) for the site-specific insertion of gene cassettes [8]. The 3′-CS of class 1 integrons typically contains the *qacEΔ1* gene, mediating low-level resistance to quaternary ammonium compounds [9], the sulfonamide resistance gene, *sul1* [10] and ORF5 of unknown function [3].

An accurate assessment of the importance of integrons in the dissemination of antibiotic resistance genes is compounded by the detection of class 1 integrons in which gene cassette regions containing antibiotic resistance genes are unable to be amplified using standard PCR methods [11]–[15]. This shortfall in standard PCR screening methods prevents the true breadth of resistance gene carriage in the community from being revealed. In this study, serologically diverse *E. coli* strains from a range of sources were screened for the presence of class 1 integrons. Cassette arrays were characterized in all of the integrons, including those that could not be amplified using standard PCR methods.

## Materials and Methods

### *E. coli* strains used in this study

512 *E. coli* strains were isolated from a variety of sources (Table 1). None of the *E. coli* strains sourced from either animals or humans were from a known outbreak. *E. coli* isolates from clinical cases of human infection were selected for screening on the basis of having resistance to at least one antibiotic. There was no selection criteria applied to the other *E. coli* strains and all strains received were screened for class 1 integrons. *E. coli* isolates from cattle and swine with clinical cases of infection had been submitted to the Elizabeth Macarthur Agricultural Institute, NSW, Australia. Canine-derived *E. coli* strains were sourced from the Faculty of Veterinary Science, University of Melbourne, Victoria, Australia. *E. coli* strains from Australian native animals were obtained from the School of Botany and Zoology, The Australian National University, ACT, Australia.

**Table 1:** *E. coli* strains examined for class 1 integron carriage.

Source of strains			Number of strains
Animal	Diagnostic specimens	Bovine	177
		Canine	46
		Porcine	20
	Healthy	Native animals	54
		Porcine	4
		Healthy sheep	1
	Unknown symptoms	Porcine	1
		Parrot	1
	**Total no. of animal-derived ** ***E. coli*** ** strains**		**304**
Human	Diagnostic specimens	UTI	95
		SIDS	21
		Diarrhoea	22
		Bloody diarrhoea	7
		Suspected diarrhoea	5
		Gastroenteritis	4
		Infantile gastroenteritis	3
		Enteritis	1
		Suspected traveler's diarrhoea/infantile enteritis	1
		Septicemia	5
		HUS	2
		Suspected HUS	1
		Neonatal meningitis	1
		Appendicitis	1
	Healthy	Infants	24
		Human	2
	Unknown symptoms	Human	7
	**Total no. of human-derived ** ***E. coli*** ** strains**		**202**
**Environmental-derived ** ***E. coli*** ** strains**			**6**
**Total number of** *E. coli* **strains**			**512**

Abbreviations: UTI, Urinary tract infection; SIDS, sudden infant death syndrome; HUS, hemolytic uremic syndrome.

Of the human-derived *E. coli* strains, 190 were collected as part of the work of the Microbiological Diagnostic Unit (MDU, Public Health Laboratory, Department of Microbiology and Immunology, University of Melbourne, Victoria, Australia) from 1994 to 2003, and 12 were archived strains from patients in the UK, USA, Denmark, Austria, Germany, Indonesia, Thailand and Japan. The 24 strains isolated from healthy infants were part of a previous study of antibiotic resistance in verocytotoxigenic *E. coli* (VTEC) and non-VTEC isolated from animals and humans [16]. These strains were collected between 1989 and 1992 from infants less than 1 year of age who had not suffered from gastrointestinal symptoms or been treated with antibiotics in the 2 weeks prior to collection.

### Characterization of *E. coli* strains

Each of the strains was identified as *E. coli* based on conformity to species description when cultured in various specialized media. *E. coli* strains were O-serotyped and H-serotyped, as previously described [16]. The plate/replicator method was used to test the sensitivity of strains to the following antibiotics: ampicillin (32 µg/ml), streptomycin (25 µg/ml), tetracycline (20 µg/ml), chloramphenicol (10 µg/ml), sulfathiazole (550 µg/ml) trimethoprim (50 µg/ml), kanamycin (10 µg/ml), nalidixic acid (50 µg/ml), spectinomycin (50 µg/ml), gentamicin (2.5 µg/ml) and ciprofloxacin (2 µg/ml) [16]. Resistance profiling, strain identification and serotyping were performed at MDU.

### PCR amplification and RFLP analysis

Bacterial DNA used as template for PCR was extracted by suspending one or two fresh colonies in 1 ml of sterile distilled water, which was subsequently washed, resuspended in 500 µl of water and heated at 100°C for 7 min. A volume of 3–5 µl of this DNA preparation was used per 50 µl PCR reaction mix. PCR targeting the *intI1* gene was performed to identify by inference, *E. coli* strains that contained class 1 integrons using the primers L2 and L3 [12]. PCR amplification of the *E. coli*-specific universal stress protein A (*uspA*) gene using the primers EC2 [17] and FD-uspA (5′ AAA GTT TCT CTG ATC CAC GTA G) served to confirm the identification of *E. coli* isolates and DNA integrity. The *E. coli* strain UB1637 (pR388, In3) was used as a positive control for PCR. The *intI1* and *uspA* PCR was performed simultaneously using the following cycling conditions: 94°C for 4 min, followed by 30 cycles of 94°C for 30 sec, 57°C for 1 min and 72°C for 1 min, and finally 72°C for 10 min. Gene cassette regions of class 1 integrons were amplified using the primers L1 and R1 [12] using conditions described previously [18]. The primer pair L1 and JL-D2 (5′ CGC ATC ACC TCA ATA CCT T) (X. Liu, unpublished results) was used to amplify integron-IS*26* elements.

PCR amplification of the *intI1* gene linked to the Tn*21* transposase genes (*tnpM*, *tnpR* and *tnpA*) was achieved using the primer pair FD-tnpA (5′ GGT CGG TAT CGT TGA ATG TGT) and FD-IntI1 (5′ GTT ACG ACA TTC GAA CCG TG). The following PCR conditions were used to amplify the integron-IS*26* gene arrangement and *intI1* linked to Tn*21* transposase genes: 94°C for 3 min, followed by 30 cycles of 94°C for 1 min, 55°C for 1 min and 72°C for 4 min, and finally 72°C for 10 min.

Gene cassette regions amplified using the standard primer pair L1 and R1 were classified according to their restriction profile, following restriction digestion with *Rsa*I and *Alu*I. RFLP analysis of the integron-IS*26* gene arrangement was performed by digestion with *Rsa*I. RFLP analysis of the *intI1*-Tn*21* amplicon was undertaken with *Eco*RI.

### Transformation studies and Southern Hybridization

In order to identify an *E. coli* isolate with the integron situated on a plasmid to facilitate DNA sequencing of the *intI1* gene and flanking sequence, transformation studies were performed. The presence of the *intI1* gene on a plasmid was determined in seven of the *E. coli* isolates (D18, D22, D79, D87, D111, D398 and D318) where cassette arrays could not be amplified using the standard primer pair L1 and R1. These *E*. *coli* isolates, with differing serotypes and resistance phenotypes, were randomly selected from the collection. Plasmid-associated *intI1* gene was detected by the electroporation of plasmid DNA isolated from these strains into *E*. *coli* JM109, followed by selection with trimethoprim (50 µg/ml), sulfathiazole (550 µg/ml), or streptomycin (25 µg/ml) and *intI1* PCR of DNA from the resultant colonies.

Southern hybridizations were also were undertaken on four *E. coli* isolates (D21, D22, D23 and D87) to identify an *E. coli* isolate located on a plasmid and facilitate DNA sequencing of the integron and flanking sequence. *E. coli* isolates D21 and D23 were randomly selected from the collection while D22 and D87 were selected from the subset of *E. coli* isolates previously tested by transformation. Southern hybridizations involved observing the hybridization of a DIG-labelled integrase probe to plasmid DNA extractions compared with that of total genomic DNA. The primers L2 and L3 [12] were used to synthesize the class 1 integrase (*intI1*) probe.

### DNA sequence analysis

Plasmid DNA isolated from the *E. coli* strain D22 was transformed [19] into *E. coli* JM109 and the DNA sequence of the integron and flanking regions of the plasmid was determined. DNA sequence analysis was performed by the dideoxy chain termination method [20] using an ABI 3130 Genetic Analyzer (Applied Biosystems, Foster City, CA, USA). The nucleotide sequence of one representative amplicon from each of the other RFLP cassette array groups described in this study was also determined.

## Results

### PCR detection of the *intI1* gene and characterization of cassette arrays

PCR screening for *intI1* identified 79/512 (15.4%) *E. coli* strains contained class 1 integrons. The *intI1* gene was detected in 32/304 (10.5%) of *E. coli* strains sourced from animals and 47/202 (23.3%) strains sourced from healthy and ill humans. The *intI1* gene was not detected in *E. coli* strains from Australian native animals or the environment.

**Figure 1:**
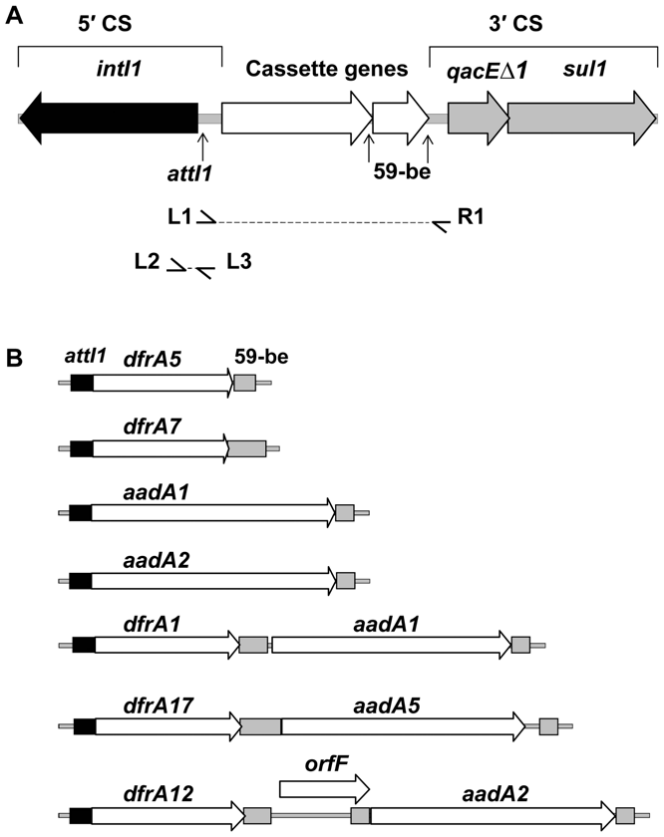
Features and characterization of typical class 1 integrons. (**A**) Structure of a typical class 1 integron showing the 5′- and 3′-CS. The location and the direction of transcription of genes are indicated. The class 1 integrase gene *intI1* (black arrow) and *attI1* integron-integration site (vertical arrow) are located in the integron 5′-CS. The *qacEΔ1* gene and the *sul1* gene (grey arrows), and the open reading frame *orf5* (not shown) are located in the 3′-CS. Inserted gene cassettes are represented by unfilled arrows and their associated 59-be are indicated by vertical arrows. L1, R1 and L2, L3 primer annealing sites are indicated by small horizontal arrows. (**B**) A diagram showing the genetic structure of the gene cassette arrays amplified using standard PCR primers that target the 5′- and 3′-CS of typical class 1 integrons (cassette arrays detected: *dfrA5*, *dfrA7*, *aadA1*, *aadA2*, *dfrA1/aadA1*, *dfrA17/aadA5* and *dfrA12/orfF/aadA2*). The following features are indicated: *attI1* recombination sites (black filled boxes), gene cassette arrays (unfilled arrows) and 59-bes (grey filled boxes). All diagrams are drawn to scale.

Gene cassette arrays within class 1 integrons were PCR amplified using standard L1 and R1 primers. RFLP identified 7 distinct RFLP groups. DNA sequence analysis of a selected representative of each RFLP group showed 100% identity to sequences previously deposited in GenBank. The following cassette arrays were identified: *dfrA5*, (AJ419169), n = 9; *dfrA7*, (EU250577); n = 2, *aadA1*, (AB188267), n = 30; *aadA2*, (DQ238100), n = 1; *dfrA1/aadA1*, (AJ884723), n = 2; *dfrA17/aadA5*, (AY748452), n = 4 and *dfrA12/*orfF*/aadA2*, (AB297450), n = 1 (Figure 1). In 31/79 integron positive *E. coli* strains, the cassette arrays could not be PCR amplified using standard L1 and R1 primers. These *E. coli* strains were sourced from cattle with clinical cases of infection (n = 27) and human patients suffering from UTI (n = 2) and bloody diarrhoea (n = 2).

### Characterization of a representative class 1 integron gene cassette array that could not be PCR amplified using standard L1 and R1 primers

**Figure 2:**
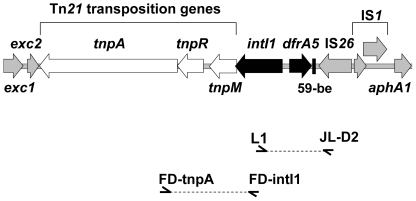
An atypical class 1 integron with an IS*26*-mediated deletion in the integron 3′-CS. Genetic structure of an atypical class 1 integron located on a plasmid isolated from the bovine-derived *E. coli* strain, D22. The genetic map represents 8756 bp of nucleotide sequence. All genes are indicated by arrows and shaded in grey, except the class 1 integron *intI1* gene and *dfrA5* cassette gene (black) and the Tn*21* transposition genes *tnpM*, *tnpR* and *tnpA* (unfilled). Other genes identified include the entry exclusion proteins *exc1* and *exc2* (truncated) and the kanamycin resistance gene, *aphA1* (partial sequence). Features involved in recombination include the 59-be (black filled box), IS*26* transposase gene, *tnpA*, and IS*1* genes *insA* and *insB*. The position of PCR primers used to screen *E. coli* strains for this atypical class 1 integron is shown.

Transformation studies revealed the integron was located on a plasmid in *E. coli* strains D18, D22 and D79 and located on chromosomal DNA in strains D87, D111, D298 and D318. Southern hybridization using the *intI1* gene as a probe demonstrated that the *intI1* gene was plasmid-borne in *E. coli* strains D21 and D22 and chromosomally located in strains D23 and D87 (results not shown). *E. coli* strain D22, containing a plasmid-associated *intI1* gene identified by Southern hybridization and transformation studies, was selected for sequencing of the integron and flanking regions. The bovine-derived *E. coli* strain, D22 contained an integron located on a plasmid of approximately 22 kb. Approximately 8.7 kb of the plasmid, encompassing the integron and flanking regions was subjected to DNA sequence analysis (GenBank accession number EU914098). This analysis revealed a class 1 integron with the typical 5′-CS and a complete *dfrA5* gene cassette, but lacked the usual class 1 integron-associated 3′-CS due to the insertion of IS*26* (Figure 2). The insertion of IS*26* presumably caused the deletion of all but 24 bp of the 3′-CS. The Tn*21* transposition genes *tnpM*, *tnpR* and *tnpA* were located downstream of the *intI1* gene.

### Distribution of *dfrA5*-IS*26* configuration and linkage of *intI1* with Tn*21* transposition genes

Identification of the unique class 1 integron-IS*26* gene arrangement in the bovine-derived strain D22 prompted the PCR interrogation of all strains with “non-amplifiable” gene cassette arrays in class 1 integrons using primers that target *intI1* and IS*26*. The presence of an integron-IS*26* gene arrangement was detected using primers L1 and JL-D2. A single 0.8 kb amplification product was detected in 27/27 *E. coli* isolates sourced from cattle and 4/4 isolates from humans. Subsequent RFLP analysis using the restriction enzyme *Rsa*I revealed the presence of the *dfrA5-*IS*26* configuration in each instance (Table 2). Nucleotide sequencing and clustalW alignment of the 848 bp amplicons in 2 human-derived and 4 bovine-derived *E. coli* strains confirmed that in each instance IS*26* was inserted in an identical position as that described for *E. coli* strain D22. Linkage of Tn*21* transposition genes and *intI1* was detected using the PCR primers FD-tnpA and FD-intI1. A single 1.5 kb PCR amplification product was detected in 18/27 *E. coli* isolates sourced from cattle and 2/4 isolates from humans (Table 2). RFLP analysis indicated these PCR amplification products were identical and nucleotide sequencing of the 1490 bp amplicon identified the integrase gene, *intI1* and the Tn*21* transposition genes, *tnpM*, *tnpR and tnpA*.

**Table 2:** Features of *E. coli* strains harbouring integron-IS*26* elements.

Strain no.	Year	Serotype	Source	A	S	T	C	Su	Tm	K	Na	G	Cp	Sp	Tn*21 tnpM, tnpR, tnpA*
D18	2002	O123:H11	B - P7	+	+	+	−	+	+	+	−	−	−	−	+
D21	2002	O123:H11	B - P7	+	+	+	−	+	+	+	−	−	−	−	+
AD22	2002	O26:H11	B - P3	+	+	+	−	+	+	+	−	−	−	−	+
D23	2002	O111:H11	B - P4	+	+	+	−	+	−	−	−	−	−	−	−
D48A	2002	O111:H11	B - P2	+	+	+	−	+	+	+	−	−	−	−	−
D49	2002	O111:H11	B - P2	+	B+	+	−	+	+	+	−	−	−	−	−
D52	2002	O111:H-	B - P2	+	B+	−	−	+	+	+	−	−	−	−	−
D53	2002	O111:H^-^	B - P2	+	B+	+	−	+	+	+	−	−	−	−	−
D55	2002	O123:H11	B - P8	+	+	+	−	+	+	+	−	−	−	−	+
D79	2002	O111:H^-^	B - P6	+	+	+	−	+	+	+	−	−	−	−	+
D81	2002	O111:H^-^	B - P6	+	+	+	−	+	+	+	−	−	−	−	+
D87	2002	O111:H11	B - P5	+	+	−	−	+	+	+	−	−	−	−	−
D101	2002	O111:H^-^	B - P1	+	+	+	−	+	+	+	−	−	−	−	+
D103	2002	O111:H^-^	B - P1	+	+	+	−	+	+	+	−	−	−	−	+
D105	2002	Ont:H9	B - P1	+	+	+	−	+	+	+	−	−	−	−	+
D111	2002	O177:H11	B - P1	−	+	+	−	+	+	+	−	−	−	−	+
D120	2002	O111:H^-^	B - P1	+	+	+	−	+	+	+	−	−	−	−	+
D252	2002	O177:H11	B - P1	−	+	+	−	+	+	+	−	−	−	−	−
D257	2002	O111:H^-^	B - P1	+	+	+	−	+	+	+	−	−	−	−	+
D260	2002	O111:H^-^	B - P1	+	+	+	−	+	+	+	−	−	−	−	+
D263	2002	O111:H^-^	B - P1	+	+	+	−	+	+	+	−	−	−	−	+
D272	2002	O111:H11	B - P5	+	+	−	−	+	+	+	−	−	−	−	−
D275	2002	O111:H11	B - P5	+	+	−	−	+	+	+	−	−	−	−	+
D298	2002	O180:H^-^	B - P9	+	+	+	−	+	+	+	−	−	−	−	+
D305	2002	O111:H^-^	B - P1	+	+	+	−	+	+	+	−	−	−	−	+
D318	2002	Ont:H32	B - P2	+	+	+	−	+	+	+	−	−	−	−	+
D319	2002	O162:H9	B - P1	+	+	+	−	+	+	+	−	−	−	−	−
20	1999	Ont:H32	H - UTI	+	−	−	−	−	+	−	−	−	−	−	−
11604	1999	O11:H^-^	H - UTI UTIUUUTI	−	−	−	−	−	+	−	−	−	−	−	−
6877	1998	O26:H^-^	H - BD	+	+	+	−	+	+	+	−	−	−	−	+
6878	1998	O26:H^-^	H - BD	+	+	+	−	+	+	+	−	−	−	−	+

A The genetic structure of the integron-*dfrA5*-IS*26* element and flanking regions of *E. coli* strain D22 is illustrated in Figure 2.

B Moderate level of resistance. Resistance to antibiotics is indicated by (+) and susceptibility is indicated by (−). Abbreviations of antibiotics: A, ampicillin (32 µg/ml); S, streptomycin (25 µg/ml); T, tetracycline (20 µg/ml); C, chloramphenicol (10 µg/ml); Su, sulfathiazole (550 µg/ml); Tm, trimethoprim (50 µg/ml); K, kanamycin (10 µg/ml); Na, nalidixic acid (50 µg/ml); Sp, spectinomycin (50 µg/ml); G, gentamicin (2.5 µg/ml); and Cp, ciprofloxacin (2 µg/ml). The source of *E. coli* strains is indicated by B, Bovine or H, human. The location of cattle properties is indicated by P1, Kameruka; P2, Cowra; P3, Eden; P4, Dungog; P5, Finley; P6, Gerringong; P7, Bega; P8, Canowindra and P9, Richmond. Human-derived *E. coli* strains were sourced from the Melbourne Diagnostic Unit (MDU) from patients with bloody diarrhoea (BD) or urinary tract infections (UTI).

### Serotypes

Features of *E. coli* strains containing the *dfrA5*-IS*26* configuration are summarized in Table 2. Variation in serotypes was observed in *E. coli* isolates from animal and human sources that contained the novel *dfrA5*-IS*26* configuration, suggesting that the horizontal transfer of this integron had occurred. Nine *E. coli* strains, with the serotype O111:H^-^ that were isolated between 2002 and 2003 and sourced from cattle geographically separated by up to 319 km, harbored the *dfrA5*-IS*26* configuration. Multiple strains with identical cassette arrays and serotypes, suggests the clonal spread of integron-containing *E. coli* had occurred between hosts.

## Discussion

In this study we have characterized antibiotic resistance gene cassette arrays in class 1 integrons that could not be amplified using standard PCR primers, by using primer pairs that target the *intI1* gene and IS*26*. Characterization of previously “non-amplifiable” cassette arrays resulted in the identification a unique *dfrA5*-IS*26* configuration in *E. coli* strains isolated from both animal and human sources. In each instance, IS*26* was inserted beyond the 59-base element of the *dfrA5* gene cassette and after the first 24 bp of the 3′-CS.

The distribution of the *dfrA5*-IS*26* configuration was widespread with regard to geographical location. The *dfrA5*-IS*26* configuration was detected in *E. coli* strains sourced from cattle located at nine NSW properties separated by up to 900 km. The distribution of the *dfrA5*-IS*26* configuration described in this study is not likely to be due to IS*26* insertion into a “hot spot” in the class 1 integron 3′-CS, as no marked target site specificity has been previously observed for IS*26* [21]. This study also established a linkage between the *intI1* and Tn*21* transposition genes, in 18/27 *E. coli* isolates from bovine sources and 2/4 isolates from human sources, which implicates Tn*21* in the distribution of this novel integron.

Variation in *E. coli* serotypes containing the *dfrA5*-IS*26* configuration supports the contention that horizontal transfer of this gene arrangement has occurred. Identification of this novel *dfrA5*-IS*26* configuration in *E. coli* strains of different serotypes that were sourced from animals and humans, suggests that the horizontal transfer of integron-related antibiotic resistance genes has occurred between these hosts. DNA sequence analysis of the 111 kb IncI1 EHEC virulence plasmid from the human-derived enterohemorrhagic *E. coli* strain, 6877, by Venturini *et al.* found the *dfrA5*-IS*26* configuration in this isolate was within a Tn*21*-derivative [22], which confirms PCR results indicating the integron-IS*26* configuration is located adjacent to Tn*21* transposition genes in this strain (Table 2). Identification of the *dfrA5*-IS*26* configuration in a virulence plasmid, highlights the possibility of co-selection of antibiotic resistance and virulence determinants under antibiotic selective pressures [22]. This *dfrA5*-IS*26* configuration, widespread in *E. coli* isolates of bovine origin and also found in *E. coli* of human origin, may act as a conduit for the transfer of integron-related resistance genes to human pathogens.

Integron-related resistance gene transfer among *E. coli* with different serotypes sourced from both humans and animals underlies the importance of developing surveillance systems and research to further define food-chain, veterinary and environmental factors involved in the spread of antibiotic-resistant bacteria to humans. Epidemiological evidence will be needed to confirm the horizontal transfer of the *dfrA5*-IS*26* configuration in *E. coli* between animal and human hosts. Information derived from antibiotic resistance surveillance studies is crucial when assessing the risks to public health and determining the impact of regulatory guidelines concerning the responsible use of antibiotics in human and veterinary medicine.
